# Anomalous origin of the right coronary artery with interarterial course: a mid-term follow-up of 28 cases

**DOI:** 10.1038/s41598-021-97917-w

**Published:** 2021-09-21

**Authors:** Francisco Albuquerque, Pedro de Araújo Gonçalves, Hugo Marques, António Ferreira, Pedro Freitas, Pedro Lopes, Mariana Gonçalves, Hélder Dores, Nuno Cardim

**Affiliations:** 1 Department of Cardiology, Hospital de Santa Cruz, Centro Hospitalar Lisboa Ocidental, Carnaxide, Portugal; 2 UNICA-Cardiovascular CT and MRI Unit, Hospital da Luz, Av. Lusíada 100, 1500-650 Lisboa, Portugal; 3 CHRC, CEDOC, NOVA Medical School, NMS, Universidade Nova de Lisboa, Lisbon, Portugal

**Keywords:** Cardiology, Imaging

## Abstract

Anomalous origin of the right coronary artery from the opposite sinus (right-ACAOS) with interarterial course (IAC) has been associated with increased risk of sudden cardiac death (SCD). Widespread use of coronary computed tomography angiography (CCTA) has led to increased recognition of this condition, even among healthy individuals. Our study sought to examine the prevalence, anatomical characteristics, and outcomes of right-ACAOS with IAC in patients undergoing CCTA for suspected coronary artery disease (CAD). We conducted a retrospective analysis of consecutive patients referred for CCTA at one tertiary hospital from January 2012 to December 2020. Patients exhibiting right-ACAOS with IAC were analyzed for cardiac symptoms and mid-term occurrence of first MACE (cardiac death, SCD, non-fatal myocardial infarction (MI) or revascularization of the anomalous vessel). CCTAs were reviewed for anatomical high-risk features and concomitant CAD. Among 10,928 patients referred for CCTA, 28 patients with right-ACAOS with IAC were identified. Mean age was 55 ± 17 years, 64% were male and 11 (39.3%) presented stable cardiac symptoms. Most patients had at least one high risk anatomical feature. During follow-up, there were no cardiac deaths or aborted SCD episodes and only 1 patient underwent surgical revascularization of the anomalous vessel. Right-ACAOS with IAC is an uncommon finding (prevalence of 0.26%). In a contemporary population of predominantly asymptomatic patients who survived this condition well into adulthood, most patients were managed conservatively with a low event rate. Additional studies are needed to support medical follow-up as the preferred option in this setting.

## Introduction

The prevalence of anomalous origin of the coronary artery from the opposite sinus (ACAOS) is low, around 1% in the general population^1–3^. ACAOS with IAC (i.e., coronary course between the aorta and the pulmonary artery) is particularly relevant given its association with sudden cardiac death (SCD), mostly in young patients undergoing high-intensity physical activity^4–7^. This association derives mainly from retrospective cohort analysis of autopsy reports for SCD^8^. In the cases where it is the only cardiac anomaly depicted in the autopsy, it is not difficult to consider ACAOS as the most probable cause. However, several authors reported a much lower risk of SCD attributed to ACAOS when compared to that reported in autopsy series^9^. This risk is even lower in patients with right-ACAOS compared to patients with left-ACAOS^10^. One major gap in knowledge regarding ACAOS relates to adequate risk stratification^8^, notably in older asymptomatic patients in whom the tradeoff between surgical risk/morbidity and the potential therapeutic benefit might be difficult to establish^10^.

During the last decade, coronary computed tomography angiography (CCTA) has become a reference tool for the diagnosis and evaluation of coronary anomalies, providing detailed three-dimensional information on the coronary origin and trajectory that cannot be easily obtained through conventional invasive coronary angiography^11,12^. With its increasing use, particularly in the setting of suspected coronary artery disease (CAD) in patients with a low-to-intermediate cardiovascular risk, higher absolute numbers of apparently healthy individuals with incidentally diagnosed ACAOS are to be expected^13^. Data on natural history of this condition is, therefore, of utmost importance. The objective of our study was to evaluate the prevalence, anatomical characteristics, management strategies and mid-term outcomes of patients with right-ACAOS with IAC in patients undergoing CCTA for suspected CAD.

## Methods

### Patient population and follow-up

**Figure 1:**
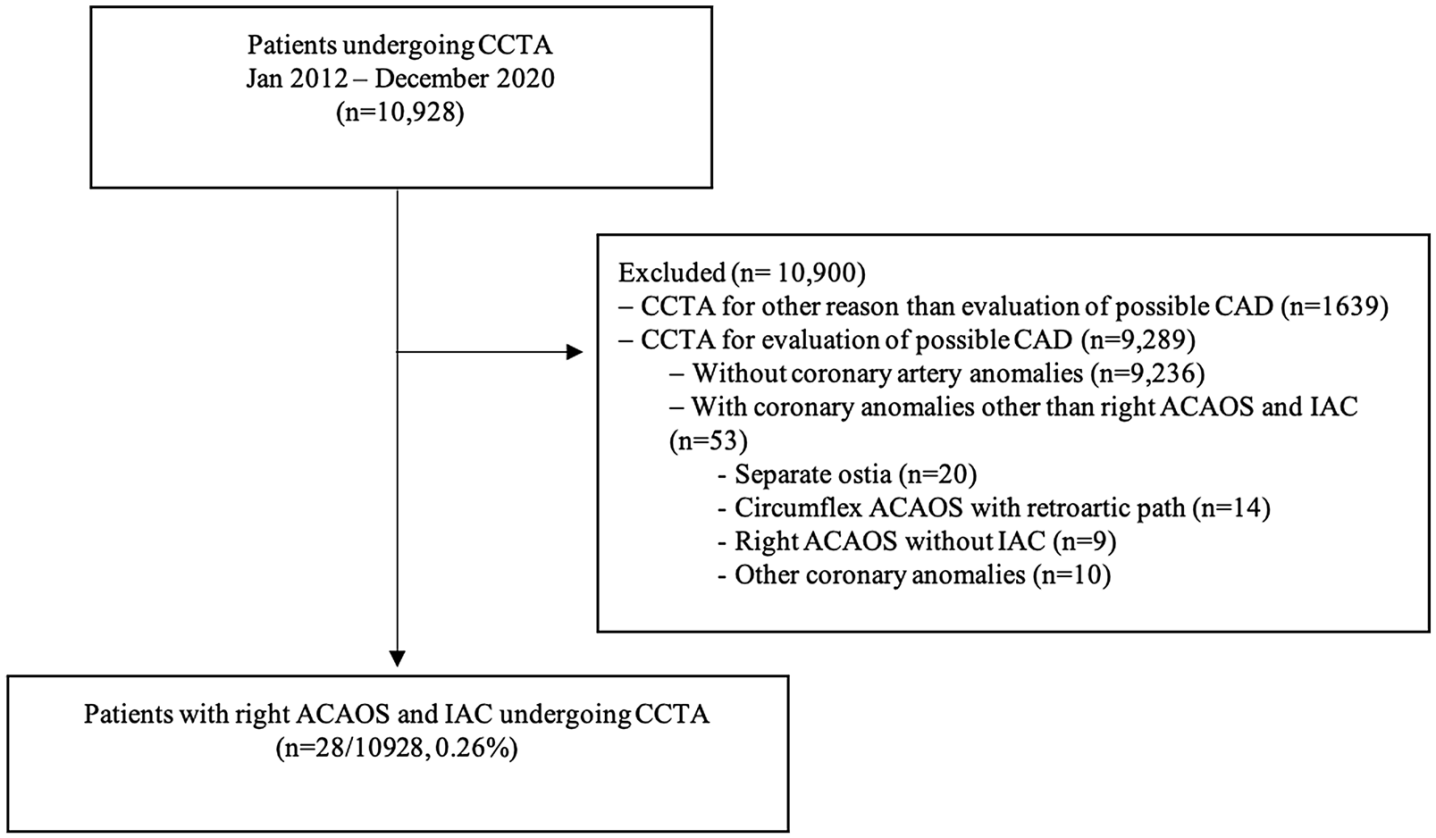
Study design. *ACAOS*  anomalous origin of the coronary artery arising from the opposite sinus; *CCTA * coronary computed tomography angiography; *IAC* interarterial course.

The study consisted of a retrospective analysis of all consecutive patients referred to contrast enhanced CCTA between January 2012 and December 2020 included in a prospective single center registry (n = 10,928). Criteria for referral to CCTA were at the discretion of the attending physician. Patients referred for reasons other than the evaluation of CAD were excluded (n = 1639, 15%, mainly evaluation for atrial fibrillation ablation and transcatheter aortic valve implantation)—Flowchart in Fig. 1. Informed consent was obtained from all patients. All methods were carried out in accordance with relevant guidelines and regulations. The study was approved by the ethics committee for health of Hospital da Luz, Lisbon, Portugal.

Baseline clinical data on demographic characteristics, cardiovascular risk factors, cardiac symptoms and previous non-invasive exams were recorded. Follow-up was performed by reviewing medical records and telephone interviews with patients and referring physicians as needed. Additionally, vital status data was also collected from medical national platform records and/or civil registries. The primary combined endpoint was the first major adverse cardiac event (MACE), defined as cardiac death, non-fatal myocardial infarction (MI) or urgent/non-urgent revascularization of the anomalous vessel (either percutaneous coronary intervention—PCI—or coronary artery bypass grafting—CABG). Cardiac death was defined as either sudden death with probable cardiac origin or death caused by acute MI, ventricular arrhythmias or refractory heart failure. Non-fatal MI was defined based on symptoms, EKG and biomarkers of ischemia.

The reports of all scans for the study period were analyzed searching for coronary artery origin anomalies and 81 patients matched this query. ACAOS with a course of the anomalous vessel between the aorta and pulmonary artery were classified as ACAOS with IAC. All ACAOS not matching these criteria were classified as ACAOS without IAC. After excluding 53 patients with coronary artery anomalies other than right-ACAOS with IAC, 28 patients were selected for analysis.

The management strategy of the patients diagnosed with right-ACAOS with IAC, including downstream complementary evaluation and/or decisions regarding revascularization, were at the discretion of the attending physician.

### CCTA evaluation

All scans were performed using a retrospective or prospective electrocardiographically gated acquisition on dual source 64-slice and 192-slice computed tomography scanners (Siemens SOMATON Definition^®^ and SOMATON Force^®^, Erlangen, Germany) according to the Society of Cardiovascular Computed Tomography Guidelines^14^. Except in the presence of conventional contraindications, sublingual nitroglycerin (0.5 mg) was administered, as were iv beta-blockers when appropriate to achieve a heart rate < 65 beats per minute.

All scans were analyzed by a cardiologist and/or radiologist with level III experience, on a dedicated postprocessing workstation (Aquarius^®^, Terarecon^®^ Inc., San Mateo, CA, USA). Abnormal findings were reviewed in a multidisciplinary team meeting.

Volume-rendered images, virtual angiographic views and double-oblique multi-planar reformatted images were used for the analysis. To ensure consistency of measurements, all CCTA studies were evaluated for the following right-ACAOS with IAC features based on previous literature^15^ and as considered appropriate by the investigators:

1. Minimum and maximum diameters: at the narrowest location and the normal distal reference segment, used to categorize proximal vessel morphology as: (i) normal, (ii) “oval” (< 50%), and (iii) “slit-like” narrowing (≥ 50% reduction in minimum diameter in the absence of coronary artery disease).
2. Length of narrowing: centerline length of vessel narrowing extending from the most proximal segment to the normal caliber distal reference.
3. Acute angle: defined as the presence or absence of acute angle take-off < 45º between (a) the plane formed by the ostium center to a point 5 mm along the vessel centerline, and (b) a plane tangent to the aorta in multiplanar axial reconstruction at the level of the right-ACAOS ostium.
4. Intramural course: defined as (i) present, (ii) absent, or (iii) indeterminate.
  Consistent with prior research^15^, an intramural course (i.e., within the aortic wall) was suspected in cases with (a) proximal vessel narrowing, (b) acute take-off (<45º), and (c) separate ostium of the vessel from the aorta. We also incorporated direct visualization of the vessel within the aortic wall (optimized by window width/level ≈ 1000/30), and the absence of adjacent epicardial fat (tissue region of interest mean signal < − 30 Hounsfield Units) for intramural course assessment, as previously described^15^.
5. Vessel take-off level: categorized as at/above or below the sinus tubular junction.
6. Ostia type: defined as (i) separate, (ii) shared, or (iii) branch vessel.

**Figure 2:**
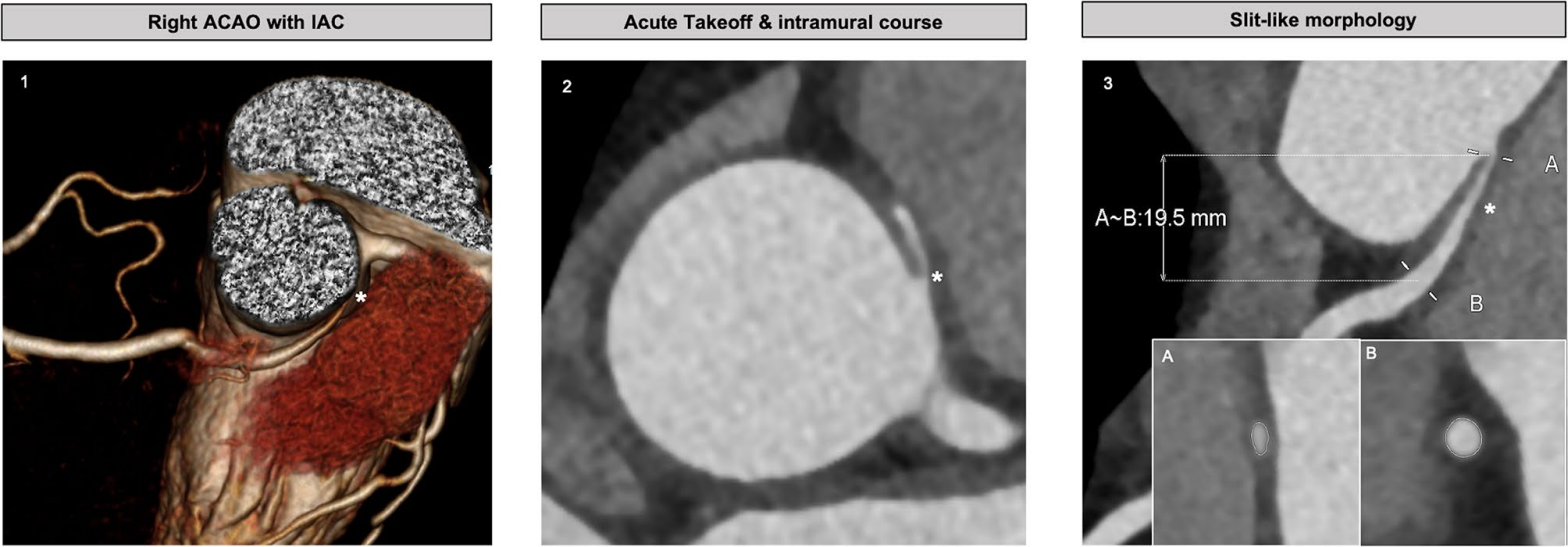
High risk anatomic features of right-ACAOS with IAC on CCTA—CCTA-identified right-ACAOS features. (1) 3D image depicting anomalous origin of the coronary artery arising from the opposite sinus with interarterial course between aorta and pulmonary artery. (2) Intramural location and take-off angles obtained in multiplanar axial reconstructions at the level of the ACAOS ostium. (3) Slit-dlike morphology. Lumen diameters obtained in double oblique view, taking the maximum and minimum diameters of the vessel at the most narrowed proximal location **(A)** and the distal reference **(B)**. A–B distance represents the interarterial course length of the anomalous vessel. *ACAOS* anomalous origin of the coronary artery arising from the opposite sinus; *CCTA* coronary computed tomography angiography; *IAC* interarterial course.

According to previous literature^13,16,17^, anatomical features such as slit-like vessel origin, acute angle take-off (< 45º), intramural course and proximal vessel narrowing of the anomalous vessel were classified as high-risk (Fig. 2).

Obstructive CAD was defined as a narrowing of the luminal diameter ≥ 50%.

### Statistical analysis

Continuous data are reported as mean and standard deviation (SD) or median and interquartile range (IQR) 25th–75th percentile, as appropriate, and categorical data as frequencies and percentages. Categorical variables were compared using chi-squared or Fisher’s exact test, and continuous variables using Student’s t-test or Mann–Whitney U-test, as appropriate.

Statistical analysis was performed using Statistical Package for Social Sciences (SPSS) version 25.0 (SPSS Inc., Chicago, IL, USA) for Mac OS. The statistical significance level was set at p < 0.05 (two-sided).

## Results

### Patient population

Among 10,928 patients referred for CCTA during the study period, of whom n = 9289 for the evaluation of possible CAD, we identified 28 patients with right-ACAOS with IAC, resulting in an overall prevalence of 0.26%. The study flow-chart is summarized in Fig. 1. Baseline characteristics are presented in Table 1. The mean age of the study population was 55 ± 17 years and the majority (18 patients, 64.3%) were male. Most patients had at least one conventional cardiovascular risk factor at baseline, hypertension being the most common. Half of the subjects had undergone a previous non-invasive assessment, with an exercise treadmill test being performed in 11 patients (39.1%). Chest pain was the most common symptom for CCTA referral (n = 8, 28.6%), followed by syncope (n = 2, 7.1%) and palpitations (n = 1, 3.6%), while 60.7% (n = 17) were asymptomatic and referred for CCTA for cardiovascular risk stratification due to positive or inconclusive non-invasive tests.

**Table 1:** Baseline characteristics of patients with right coronary artery anomalous origin and an interarterial course.

	All patients (n = 28)	Young patients ^a^ (n = 4)	High-risk^a^ (n = 22)^c^	No high-risk (n = 6)	p-value
Age, years, mean ± SD	55 ± 17	^b^ 25 (18–29)	54 ± 17	59 ± 21	0.561
Male, n (%)	18 (64.3)	2 (50.0)	13 (59.1)	5 (83.3)	0.272
Hypertension, n (%)	18 (64.3)	0 (0.0)	13 (59.1)	5 (83.3)	0.272
Hyperlipidaemia, n (%)	17 (60.7)	1 (25.0)	13 (59.1)	4 (66.7)	0.736
Diabetes mellitus, n (%)	5 (17.9)	0 (0.0)	4 (18.2)	1 (20.0)	0.932
Current or prior smoker, n (%)	2 (7.1)	0 (0.0)	2 (9.1)	0 (0.0)	0.443
Family history premature CAD, n (%)	5 (17.9)	1 (25.0)	5 (22.7)	0 (0.0)	0.198
Known CAD, n (%)	3 (10.7)	1 (25.0)	2 (9.1)	1 (16.7)	0.595
**Indication for evaluation with CCTA, n (%)**					
Asymptomatic with positive ischemia or inconclusive ischemia test Cardiovascular symptoms	17 (60.7) 11 (39.3)	2 (25.0) 2 (50.0)	15 (68.2) 7 (31.2)	2 (33.3) 4 (66.7)	0.634 0.634

Values are mean (SD) or n (%).

*CAD* coronary artery disease, *CCTA* coronary computed tomography angiography, *SD* standard deviation.

^a^Young patients representing patients < 30 years old.

^b^Mean, minimum and maximum age, respectively.

^c^High-risk defined as the presence of at least one of the following features: (i) slit-like proximal vessel morphology; (ii) acute take-off angle; (iii) presence of an intramural segment.

### CCTA findings

Details on right-ACAOS with IAC characteristics including high-risk anatomic features on CCTA are depicted in Table 2. Most right-ACAOS with IAC had an origin from a separate ostium and above the sinotubular junction. An intramural segment was present in 9 (32.1%) patients. The median length of narrowing was 21 (11–25) mm. Most patients (n = 23; 82.1%) had right-ACAOS originating from the left cusp and 5 (18.9%) from a shared ostium with left main (supplementary Fig. 1).

**Table 2:** Anatomic features of right-ACAOS with IAC on CCTA.

	All patients (n = 28)
**Proximal vessel morphology, n (%)**	
Oval (< 50% narrowing) ^a^Slit-like (≥ 50% narrowing)	18 (64.3) 10 (35.7)
Length of narrowing, mm	20.9 (10.5–24.5)
^a^Acute take-off angle < 45º, n (%)	22 (78.6)
**Intramural segment, n (%)**	
Not present ^a^Present	19 (67.9) 9 (32.1)
Take-off level above STJ, n (%)	18 (64.3)
**Take-off type**	
Separate ostia Shared ostia	23 (82.1) 5 (17.9)
Any CAD > 50% stenosis, n (%)	3 (10.7)

*CAD* coronary artery disease, *CCTA* coronary computed tomography angiography, *IAC* interarterial course, *STJ* sinotubular junction.

^a^High risk features according to published literature.

According to previous literature, the majority of patients (78.6%, n = 22) had at least one anatomical feature considered to be high risk. Anatomical characteristics of the patient population on CCTA are presented in Table 2 and Fig. 2. Only 3 (10.7%) patients had concomitant obstructive coronary artery disease (2 patients in the left coronary artery and 1 patient in the distal course of the right—ACAOS).

### Outcomes

All patients were included in the outcome analysis as no patient was lost during follow-up. The median overall follow-up was 45.5 (10.6–70.8) months. During the follow-up period, no deaths were observed, and a first MACE occurred in only 1 (3.6%) patient. This patient underwent non-urgent right-ACAOS-related cardiac surgery, with CABG of the anomalous vessel. The patient was referred to CCTA due to anginal chest pain, had a positive pre-operative ischemic test, and no concomitant obstructive CAD or intramural segment were present. The procedure and follow-up were uneventful. All other patients had a conservative management strategy, and no events were observed during follow-up.

## Discussion

The main findings of our study can be summarized as follows: (1) among patients referred for CCTA for possible CAD, the prevalence of right-ACAOS with IAC was 0.26%; (2) there were no major clinical events over a median follow-up of 45.5 months and only one patient was submitted to cardiac surgery, even despite that the majority of patients would have been classified as high-risk according to proposed anatomical findings on CCTA.

The prevalence of 0.26% for right-ACAOS with IAC is in line with previous published literature^8^. The majority were middle-aged patients with low to intermediate pretest probability of obstructive CAD, a population where increased rates of incidental diagnosis of ACAOS are expected because of the expanding use of CCTA for the exclusion of CAD in this subgroup of patients^12,18^.

Many cases of coronary anomalies are asymptomatic at the time of clinical evaluation or diagnosis^4,5,10,19–22^. Although chest pain was the most common symptom for CCTA referral in our study, right-ACAOS with IAC was probably an incidental diagnosis in most cases evaluated, a finding in line with previous reports^8^. Furthermore, published literature suggests that mild cases are more likely to be identified fortuitously^23^, which may have been the case in our study given the high percentage of patients undergoing CCTA due to a false positive treadmill test. The fact that these patients are often asymptomatic and may initially exhibit SCD makes their management challenging^24^.

CCTA is considered the primary imaging modality to detect and evaluate the anatomy of ACAOS^1^, offering detailed characterization with high spatial and temporal resolution. Moreover, it has the potential to identify “high-risk” anatomic features that may be useful to stratify patients and guide management. The majority of the patients in our study had at least one high-risk anatomical feature (i.e., slit like origin, acute angle take-off, an intramural segment).

Published data associating ACAOS with IAC to SCD derives largely from studies^3–5,12,24,25^: (1) analyzing retrospective autopsy data; (2) analyzing young subjects undergoing high-intensity physical activity. However, we must be wary of conclusions drawn from autopsy studies, which are often taken as evidence of the lesion’s high risk. Our results indicate that the absolute risk of sudden death is probably low, as has already been reported^9^. There were no deaths during the follow-up period and only one patient underwent non-urgent right-ACAOS with IAC-related cardiac surgery, with CABG of the anomalous vessel. Decision-making regarding surgery must take into account the patient’s age, the presence of symptoms, evidence of myocardial ischaemia or high-risk anatomy according to the 2020 ESC Guidelines for the management of adult congenital heart disease^26^. All other patients had a conservative strategy and no events were observed. Furthermore, there is evidence that a large proportion of middle-aged individuals with newly diagnosed ACAOS by CCTA are involved in sporting activities before diagnosis as well as during follow-up, regardless of whether they were surgically corrected or not, with a low rate of adverse cardiac events during follow-up^27^.

Further considerations should be highlighted regarding our results: (1) the mean age of our population is considerably higher when compared to that reported in autopsy studies; (2) most SCD cases associated with ACAOS occur in young patients performing high-intensity physical activity, which contrasts with our population of asymptomatic patients in which right-ACAOS with IAC was most probably an incidental finding. However, a recent study by Finocchiaro et al.^28^ reports that SCD associated with right-ACAOS with IAC occurred often at rest or during sleep. (3) We limited our analysis to right-ACAOS with IAC because its association with SCD is not as convincing as left-ACAOS, resulting in even more uncertainty regarding the management of this subgroup. According to one of the largest series on this issue^19^, all cases of SCD associated to ACAOS occurred in patients with left-ACAOS; (4) in a substantial proportion of our population (n = 5; 19.2%), we found a left dominant coronary circulation. Autopsy data^29^ suggests that coronary dominance, not ostial shape, is useful in identifying clinically significant anomalies and showed that when right-ACAOS with IAC is associated with left coronary dominance the anomaly is not clinically significant; (5) the low number of events found in our population occurred despite the high number of patients having at least one anatomical high-risk feature on CCTA.

### Study limitations

The present investigation has a number of limitations that should be acknowledged. First, it is a retrospective, single center study subject to inherent biases of study design and limitations on the quality of registry data. Patients were mostly referred for CCTA due to suspected CAD (symptoms and/or positive/inconclusive non-invasive tests), which might introduce a selection bias towards a higher pretest probability of CAD than a general population screening. We only included patients with right-ACAOS with IAC, excluding other types of coronary anomalies perceived to have higher risk of SCD such as left-ACAOS. The low number of events in our cohort has to consider the possible selection bias of age, since higher risk phenotypes might have been identified in younger ages or had SCD and are therefore not represented in this population. Furthermore, after diagnosis, patients may have been counselled to avoid physical activity and triggering factors, possibly reducing the incidence of MACE. CCTA findings such as high coronary calcium or non-obstructive CAD may have contributed to optimization of preventive medical therapies for atherosclerotic disease, further reducing the risk of MACE. Finally, the sample size was small with low confirmatory power given that right-ACAOS with IAC is a rare condition, and the incidence of cardiac events was extremely low.

## Conclusions

Right-ACAOS with IAC is an uncommon finding, with an observed prevalence of 0.26%. In our population of middle-aged individuals referred for CCTA for the evaluation of possible CAD, most cases of right-ACAOS with IAC were discovered fortuitously, mid-term outcome seems to be favorable with a very low risk of cardiac events and the vast majority of patients underwent conservative management. Additional studies are needed to support medical follow-up as the preferred option in this setting.

## Supplementary Information

Supplementary Figure S1.

## Data Availability

The datasets generated and analyzed during the current study are available from the corresponding author on reasonable request.
