# Supplementary figures and images for: Anomalous origin of the right coronary artery with interarterial course: a mid-term follow-up of 28 cases
Source: Sci Rep. 2021 Sep 21;11:18666. doi: 10.1038/s41598-021-97917-w (PMC8455645; doi:10.1038/s41598-021-97917-w)

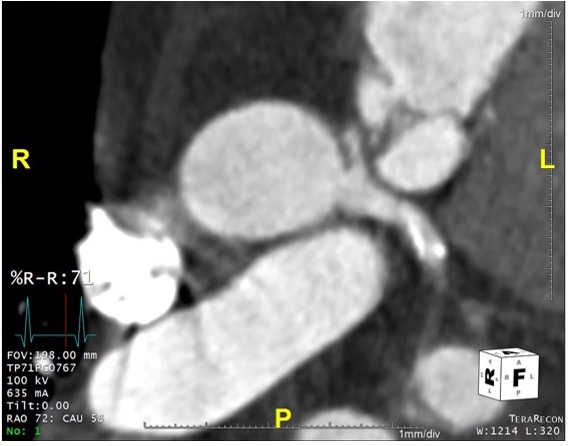

Supplement: Supplementary file 1 — Supplementary Figure S1. [file 41598_2021_97917_MOESM1_ESM.tiff]
